# Cutaneous Adenoid Cystic Carcinoma with Perineural Invasion Treated by Mohs Micrographic Surgery—A Case Report with Literature Review

**DOI:** 10.1155/2010/469049

**Published:** 2010-04-07

**Authors:** Yaohui G. Xu, Molly Hinshaw, B. Jack Longley, Humza Ilyas, Stephen N. Snow

**Affiliations:** Department of Dermatology, University of Wisconsin-Madison, One South Park St., 7th Floor, Madison, WI 53715, USA

## Abstract

We report a 58-year-old woman with cutaneous adenoid cystic carcinoma arising on the chest treated with Mohs micrographic surgery. The patient remained tumor-free at 24-month follow-up. To date, only six other cases of cutaneous adenoid cystic carcinoma were reportedly managed by Mohs surgery. Cutaneous adenoid cystic carcinoma has low potential for distant metastasis but is notorious for its aggressive infiltrative growth pattern, frequent perineural invasion, and high risk of local recurrence after excision. We propose that Mohs surgery is an ideal method to achieve margin-free removal of cutaneous adenoid cystic carcinoma. A brief literature review is provided.

## 1. Introduction

Adenoid cystic carcinoma (ACC) is commonly known as a malignant neoplasm of salivary glands in the head and neck region [1]. Rarely, ACC arises directly in the skin, for which wide local excision is the standard treatment. Cutaneous ACC has low potential for distant metastasis but is locally aggressive. Perineural invasion has been reported in 76% of cases with a local recurrence of 44% following traditional surgical excision [2], warranting a better management modality. We propose that a margin-free excision achieved by Mohs surgery is theoretically superior to traditional wide local excision. A brief literature review with regard to clinical presentation, histopathology, and treatment of cutaneous ACC is provided.

## 2. Case Report

A 58-year-old, otherwise healthy, Caucasian female was referred to the Department of Dermatology at the University of Wisconsin for treatment of a biopsy proven adenoid cystic carcinoma on the right mid-chest. She had a lesion of “basal cell carcinoma” surgically excised on her right mid-chest 14 years previously. The actual slides of the previous excision were not retrievable, while the pathology report did state that the lesion was “a basal cell carcinoma which in some areas presents gland-like formations, adenoid cystic characteristics and focal areas of keratinization, but it predominantly is an adenoid basal cell carcinoma.” The patient had done well until one year prior to the referral when she noted two tender, flesh-colored nodules adjacent to the scar. A punch skin biopsy confirmed the diagnosis of adenoid cystic carcinoma concerning for its infiltrative growth pattern and perineural invasion. 

**Figure 1:**
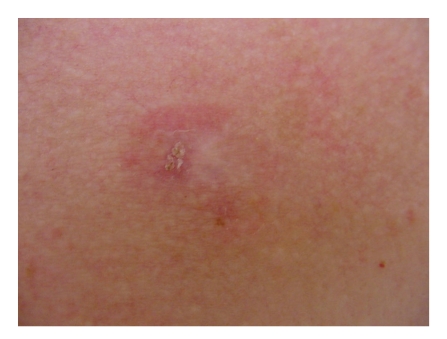
Clinical morphology prior to the Mohs surgery for a biopsy-proven ACC: A 58-year-old woman with a poorly defined erythematous patch on the mid-chest, measuring about 15 mm approximately. Note focal crust and a hypopigmented scar within the patch. An adenoid BCC of the right chest was treated by excision 14 years earlier. ACC: adenoid cystic carcinoma, BCC: basal cell carcinoma.

A thorough preoperation physical examination revealed a healthy-appearing Caucasian female with an approximately 15 × 15 mm, poorly defined, tender, erythematous patch with focal crust and a hypopigmented scar notable on the right mid-sternum region (Figure 1). There was no lymphadenopathy or organomegaly. 

**Figure 2:**
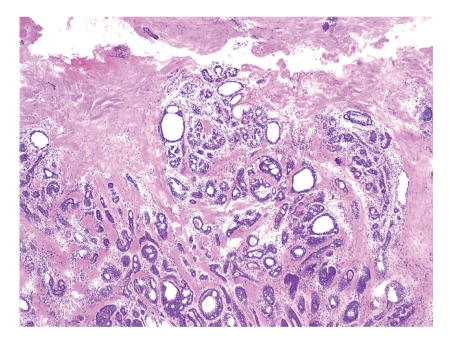
Microscopic finding of typical ACC characterized by multiple glandular and ductal structures in the deep dermis. There are multiple cysts with some being clear and others with eosinophilic materials (Fresh frozen tissue, H & E, original magnification ×40). ACC: adenoid cystic carcinoma.

**Figure 3:**
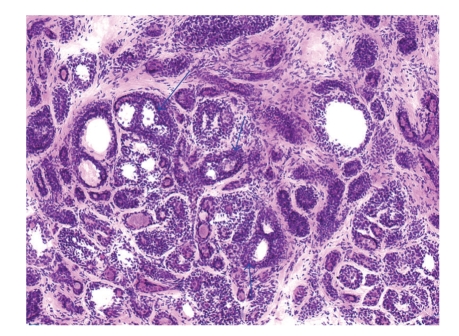
High magnification demonstrating characteristic cribriform pattern in which multiple cysts are embedded in an island of basaloid cells. Three representative cysts are shown by arrows (Fresh frozen tissue, H & E, original magnification ×100).

**Figure 4:**
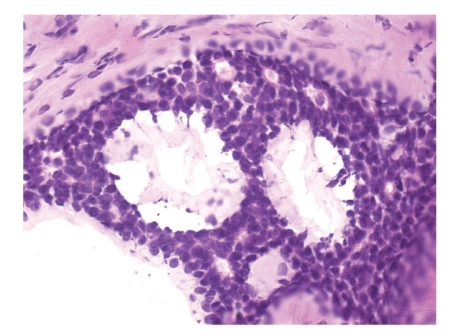
The cyst lining showing cytoplasmic blebbing reminiscent of apocrine-like decapitation secretion within the cyst (Fresh frozen tissue, H & E, original magnification ×400).

**Figure 5:**
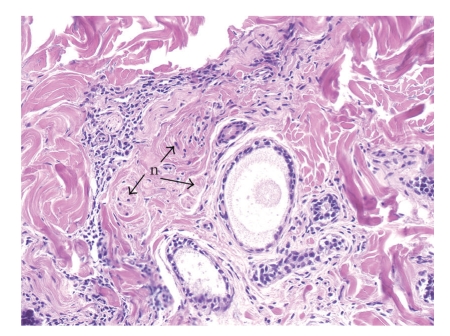
Several foci of ACC are situated adjacent to nerves (n) as indicated by arrows (Paraffin-embedded tissue, H & E, original magnification ×200). ACC: adenoid cystic carcinoma.

The patient underwent Mohs surgery for excision of the tumor. The specimens were processed using fresh frozen tissue stained with hematoxylin and eosin. It revealed an infiltrative dermal tumor composed of basaloid cells arranged in cords, nodules, and cribriform islands embedded in a fibrous stroma, with a lack of continuity with the surface epidermis. Prominent glandular and ductal differentiation and cystic spaces containing mucinous materials, and eosinophilic globules of amorphous material were noted (Figure 2). The characteristic cribriform pattern was better appreciated at a higher magnification (Figure 3). Interestingly, the cells lining the cyst showed cytoplasmic blebbing reminiscent of apocrine-like decapitation secretion (Figure 4). The specimens were also submitted for permanent sections, which clearly demonstrated several foci of basaloid tumor cells situated adjacent to nerves (Figure 5). A tumor-free plane was achieved in two stages down to the subcutaneous fat with a final defect of 31 × 24 mm, and the defect was closed primarily. Adjuvant radiation was not given.

To further rule out an extracutaneous primary site of the tumor and search for evidence of metastasis, additional work-up was completed including a breast examination, mammogram, computed tomographic (CT) scans of the head, neck, chest, and abdomen, and head and neck examination by an otolaryngologist. All were within normal limits. The patient is now 24 months postoperative, and there is no sign of local recurrence or distant metastasis. 

The authors acknowledge that for our patient, the Mohs excision of the tumor preceded the work-up for possible extracutaneous sources. The work-up was slightly delayed due to prior-authorization processes involved with her insurance company. We wish to emphasize that ideally it is advisable to complete the entire work-up prior to excision, in order to exclude any extracutaneous source and thus confirm the diagnosis of primary cutaneous ACC.

## 3. Discussion

ACC is most commonly seen as a neoplasm of the major and minor salivary glands. It can also arise from a variety of primary sites, including the external auditory canal, lacrimal gland, respiratory tract, uterus, cervix, vulva, breast, thymus, prostate, esophagus, and skin [3, 4]. When ACC arises directly in the skin, it is considered primary cutaneous ACC, to be differentiated from a metastasis or direct extension of a salivary gland ACC to the skin. Only primary cutaneous ACC is relevant to our discussion, therefore, if not otherwise specified, we will use the term cutaneous ACC for simplicity. Cutaneous ACC is a rare tumor with histological features closely resembling ACC of the salivary glands. Therefore, cutaneous and extracutaneous ACC can only be distinguished based on clinical grounds and the diagnosis of cutaneous ACC can only be established by a lack of any history or current evidence of ACC from an extracutaneous source. This has paramount significance as the ACC of salivary glands is an aggressive tumor in which local recurrence and widespread metastases result in death in the majority of patient; whereas cutaneous ACC tends to run an indolent course despite a high tendency for local recurrence [2]. 

Cutaneous ACC was first reported by Boggio in 1975 [5]. To date, just over 50 cases have been published in the English language literature. According to a recent review paper by Naylor et al. [2], the mean reported age of onset is 59 years, with a slight male predominance (male 57%). Excluding the external auditory canal, the scalp is the most common location, accounting for 41% of all cases. Other locations include the chest, abdomen, back, eyelid, and perineum. Cutaneous ACC often presents as a firm, slow-growing, ill-defined nodule or tumor that may be asymptomatic, or with symptoms including tenderness, pruritus, and secondary alopecia. Perineural invasion, a risk factor for recurrence, is seen in 76% of cases. Recurrence is documented in 44% of cases treated by wide local excision with an average follow-up length of 58 months. Distant metastasis is rare. If metastasis does occur, lymph nodes and lung are the sites of predilection. 

Microscopic findings of cutaneous ACC are characterized by basaloid cells in the mid to deep dermis, arranged in cords and islands forming tubular structures and cribriform patterns, usually with a lack of connection to the overlying epidermis or adnexal structures [6, 7]. There may be small cystic spaces containing mucinous material that stains positively for hyaluronic acid. The lumina of the tubular structures and the surrounding stroma may contain mucin or eosinophilic necrotic cells. True lumina are surrounded by prominent basement membrane material, which is PAS positive, diastase-resistant [6, 7]. Perineural invasion is often seen [6, 7].

Whether cutaneous ACC is a tumor of eccrine or apocrine origin has not been determined. There is some observational evidence suggesting that this tumor might be apocrine in origin. ACC often arises from ceruminous glands of the external ear canal, which are modified apocrine glands. 

Histologically, cutaneous ACC must be differentiated from adenoid basal cell carcinoma (BCC). Adenoid BCC also may demonstrate a cribriform pattern with areas of cystic degeneration and abundant mucin. However, it can be differentiated from cutaneous ACC by its continuity with the epidermis or adjacent hair follicle, the presence of peripheral palisading and retraction artifact, and often the lack of perineural invasion. Immunohistochemical studies may assist in further differentiating adenoid BCC from ACC. For instance, in contrast to adenoid BCC, cutaneous ACC often stains positively for S-100, epithelial membrane antigen (EMA) and is variably positive with carcinoembryonic antigen (CEA) [2, 3, 8]. Our patient had a lesion diagnosed as an adenoid BCC on the chest treated many years earlier at an outside clinic but no tissue blocks were available for further review. Therefore, we favor but cannot confirm that the lesion was an ACC from the beginning. 

Another close mimicker of cutaneous ACC is mucinous carcinoma of the skin which typically shows islands of basaloid eccrine cells embedded in lakes or pools of mucin separated by fibrous septa [9–12]. The mucin is hyaluronidase resistant and sialidase labile, indicting that it is a sialomucin, as apposed to the hyaluronic acid seen in cutaneous ACC. It is worth mentioning that there is a similar term of “adenocystic carcinoma” that is an older terminology referring to mucinous carcinoma of the skin [11, 13], but not adenoid cystic carcinoma. The use of this terminology is not recommended since it adds more confusion to the classification of sweat gland tumors [14].

Histological differential diagnosis of ACC should also include primary cutaneous cribriform apocrine carcinoma, which is a rare, low-grade cutaneous apocrine carcinoma. Microscopically primary cutaneous cribriform apocrine carcinoma is a nonencapsulated dermal tumor with an extensive cribriform pattern formed by multiple interconnected basophilic epithelial cells that are arranged in solid nests or tubular structures, and many small round spaces in between [15, 16]. As opposed to cutaneous ACC, basophilic aggregations as well as spaces within are often more varied in size and shape, cells are more interconnected, true elongated tubules, but no deposition of basement membrane material, are observed, and neoplastic cells contain pleomorphic rather than monomorphous nuclei. No perineural or intravascular invasion is seen. No metastatic potential or postexcision recurrence has been associated with this tumor, which sets it apart from more aggressive cutaneous ACC [16]. 

The standard treatment for cutaneous ACC is wide local excision with tumor-free margins established by permanent sections [3, 7]. Adjuvant therapy with local radiation is utilized by some authors [17], although no studies have demonstrated that radiation decreases the risk of local recurrence. Chemotherapy has been used in patients with distant metastases [17, 18]. 

Only 7 cases [19–24], including the current case, have been managed by Mohs surgery (Table 1), accounting for 14% of total reported cases. Cutaneous ACC has low potential for distant metastasis while locally being infiltrative with frequent perineural invasion, making it an ideal indication for Mohs surgery. The high local recurrence rate of 44% following traditional wide excision suggests incomplete removal of the tumor at the initial excision, despite negative margins established by routine histology. Discontinuous perineural extension is seen in cutaneous ACC, which may contribute to false-negative margins leading to a high recurrence rate [25, 26]. Experience with other more common cutaneous malignancies that may also exhibit perineural invasion, such as squamous cell carcinoma and basal cell carcinoma, has demonstrated the increased sensitivity in the detection of perineural invasion and the lower rates of recurrence using Mohs surgery when compared to traditional surgical excision [27–29], leading us to propose that Mohs surgery should be the treatment of choice for cutaneous ACC. 

It is worth clarifying the term “discontinuous perineural extension.” While tumors normally spread in a continuous manner, they may spread asymmetrically along the nerve. Also, tissue manipulation for horizontal sections during Mohs surgery may distort a nerve and result in “skip-areas” [25]. Therefore, when tumors invade nerves, Mohs surgery has its limitation in identifying perineural invasion conclusively due to possible false “skip areas” or the so-called “discontinuous perineural extension.” Nevertheless, Mohs surgery, by examining 100% of the surgical margin utilizing horizontal sections, still has increased sensitivity as compared to routine histological processing, in which equivalent to 0.01% of the specimen surface area is evaluated [30, 31]. As perineural inflammation and “skip areas” due to tissue processing may indicate proximal perineural invasion, some authors advocate the removal of an additional Mohs layer after tumor-free margins are obtained [32, 33]. We would like to share our experience in managing cutaneous malignancies with perineural invasion in general. If there is significant perineural invasion then an additional Mohs layer is helpful to obtain a double negative confirmation. Also, if there are pre-existing “skip areas” noted on the Mohs excision, then an additional layer needs to be considered. We recommend local radiation therapy only as a last resort when it is not possible to surgically remove the malignant tumor, because wound healing after local radiation therapy is poor. 

Some authors have questioned whether routine hematoxylin and eosin staining of fresh frozen sections from Mohs surgery provides optimal visualization of tumor margins in the treatment of cutaneous ACC. Lang et al. preferred to use permanent sections instead [19]. Given that cutaneous ACC is rich in mucin, Chesser et al. proposed to use fresh frozen sections stained with toluidine blue, which stains mucin metachromatically, to better define tumor margins [20]. In our hands, fresh frozen sections stained with routine hematoxylin and eosin seem to provide sufficient microscopic accuracy as shown in Figures 2–4. Nevertheless, we submitted tissue for permanent sections for reconfirmation. 

Based on the nature of cutaneous ACC and the success rate of Mohs surgery in other cutaneous malignancies that exhibit perineural invasion, we propose that Mohs surgery is superior to wide local excision to achieve a more complete excision of this tumor and to reduce the rate of local recurrence. In the seven cases treated by Mohs surgery, there has been no local recurrence with a follow-up range of 10–28 months. However, a limited case number and insufficient follow-up period does not allow us to draw a definitive conclusion. We hope to see more cases managed by Mohs surgery, so that one might be able to compare whether there is indeed a lower recurrence rate in cases treated with Mohs surgery as opposed to wide local excision. Additionally, since recurrence of cutaneous ACC can occur as late as 35 years postexcision [2], all patients with cutaneous ACC deserve life-long follow-up for possible recurrence. Finally, we propose that when surgically removing an adenoid BCC, all cancerous tissue are submitted for permanent sections for special immunostains to rule out the possibility of ACC or mucinous carcinoma of the skin.

## Figures and Tables

**Table 1:** Cases of primary cutaneous adenoid cystic carcinoma treated with mohs micrographic surgery.

Ref.	Sex/age	Duration	Presentation	Location	Tissue processed	PNI	LN	Adjuvant therapy	F/U (m)
[17]	F/68	Sx for ACC 5 yrs ago	Lump in a scar	Back	Permanent	(−)	(−)	None	18
[18]	M/65	1 yr	Lump	Scalp	Fresh frozen*	(−)	(−)	None	10
[19]	F/45	Sx for ACC 2 & 7 yrs ago	Tender scar	Scalp	Fresh frozen	(−)	(−)	None	18
[20]	F/57	1 yr	Nodule	Scalp	Fresh frozen	(−)	(−)	Local rad	28
[21]	F/62	Months	Painless nodule	Scalp	NS	(+)	(−)	None	6
[22]	M/59	1 yr	Asymptomatic nodule	Eyelid	NS	(+)	(−)	None	12

Our case	F/58	Sx for BCC 14 yrs ago	Tender nodules in a scar	Chest	Fresh frozen and permanent	(+)	(−)	None	24

AAC: adenoid cystic carcinoma; BCC: basal cell carcinoma; F: female; F/U: follow-up; LN: lymph node; M: male; m: months; NS: not stated; PNI: perineural invasion; rad: radiation; Ref: reference; Sx: surgery; yrs: years; *: Stained with toluidine blue technique.
